# The complete chloroplast genome sequence of *Abies chensiensis* (Pinaceae)

**DOI:** 10.1080/23802359.2018.1542992

**Published:** 2019-10-01

**Authors:** Lei Su, Peng-Fei Zhao, Xiao-Fang Lu, Yi-Zhen Shao

**Affiliations:** a School of Life Sciences, Henan University, Kaifeng, China;; b College of Life Sciences, Henan Agriculture University, Zhengzhou, China

**Keywords:** *Abies chensiensis*, chloroplast genome, threatened, phylogenetic

## Abstract

*Abies chensiensis* is listed as a threatened species in the Red List and categorized as key protected wild plants in China. Here, we determined the complete chloroplast genome of *A. chensiensis* using the Illumina MiSeq platform. The genome was 121,795 bp in length, comprising a large single copy (LSC) region of 67,160 bp, a small single copy (SSC) region of 54,107 bp, and two inverted repeat regions (IRa and IRb) of 264 bp each. It was composed of 114 genes, including 68 peptide-encoding genes, 35 transfer RNAs (tRNAs), four ribosomal RNAs (rRNAs), six open reading frames and one pseudogene. Phylogenetic analysis revealed that *A. chensiensis* was most closely related to *A. beshanzuensis*, with high bootstrap values. The present research will provide potential genetic resources for further conservation and management strategies.

*Abies chensiensis* Tiegh. is endemic in central China, with a disjunctively distribution in Henan, Shanxi, Hubei and Gansu provinces (Liu 1971; Shao and Xiang 2015). Because of its rare and narrow distribution, it is listed as a threatened species in the Red List (IUCN 2018) and categorized as key protected wild plants in China. At present, *A. chensiensis* occurs in high mountain ranges between 2100 m and 3000 m, with a cold and moist climate (Farjon 2001; Shao et al. 2017). Here, we assembled and characterized the complete plastome of *A*. *chensiensis*. It will provide potential genetic resources for further conservation and management strategies.

A strain sampled from the Shennongjia Mountain of Hubei province was used for sequencing. Specimens were given identification numbers and registered in the herbarium of Institute of Botany, CAS (PE), with Voucher no.WR0016. The complete chloroplast genome was sequenced by HiSeq4000 of Illumina. Totally 10.3 million high-quality clean reads (150 bp PE read length) were obtained. In total, ca. 10.1 million high-quality clean reads (150 bp PE read length) were generated with adaptors trimmed. The CLC de novo assembler (CLC Bio, Aarhus, Denmark), BLAST, GeSeq (Tillich et al. 2017), and tRNAscan-SE v1.3.1 (Schattner et al. 2005) were used to align, assemble, and annotate the plastome.

The chloroplast contigs of *A. chensiensis* were de novo assembled from low-coverage whole-genome sequences. The complete chloroplast genome is 121,795 bp in length (GenBank Accession no. MH706706) and has a typical quadripartite structure, consisting of a large single copy (LSC) region of 67,160 bp, a small single copy (SSC) region of 54,107 bp, and two inverted repeat regions (IRa and IRb) of 264 bp each. The GC content is 38.3%, with the SSC region having higher GC content (39.3%) than the LSC (37.4%) and IR (39.0%) regions. The genome contains 114 genes, including 68 peptide-encoding genes, 35 transfer RNAs (tRNAs), four ribosomal RNAs (rRNAs), six open reading frames and one pseudogene. All ndh genes have been lost in the genome of *A*. *chensiensis*. Short inverted repeat sequences were detected in 52-kb inversion points of the cp genome, which consist of trnS-psaM-ycf12-trnG and trnG-ycf12-psaM-trnS (1183 bp). Interestingly, such missing of ndh genes and inverted repeats had been reported in several members of the genus *Abies* (*A*. *beshanzuensis*, *A. koreana*) (Yi et al. 2015; Shao et al. 2018) .

To investigate the phylogenetic position of *Abies chensiensis*, we aligned ten chloroplast genomes with MAFFT v7.3 (Suita, Osaka, Japan) (Katoh and Standley 2013). The maximum likelihood (ML) inference was performed using GTRþIþC model with RAxML v.8.2.1 (Karlsruhe, Germany) (Stamatakis 2014) on the CIPRES cluster service (Miller et al. 2010). The ML tree revealed that *A. chensiensis* and *A. beshanzuensis* formed a monophyletic group with bootstrap support values of 100% (Figure 1).

**Figure 1.**
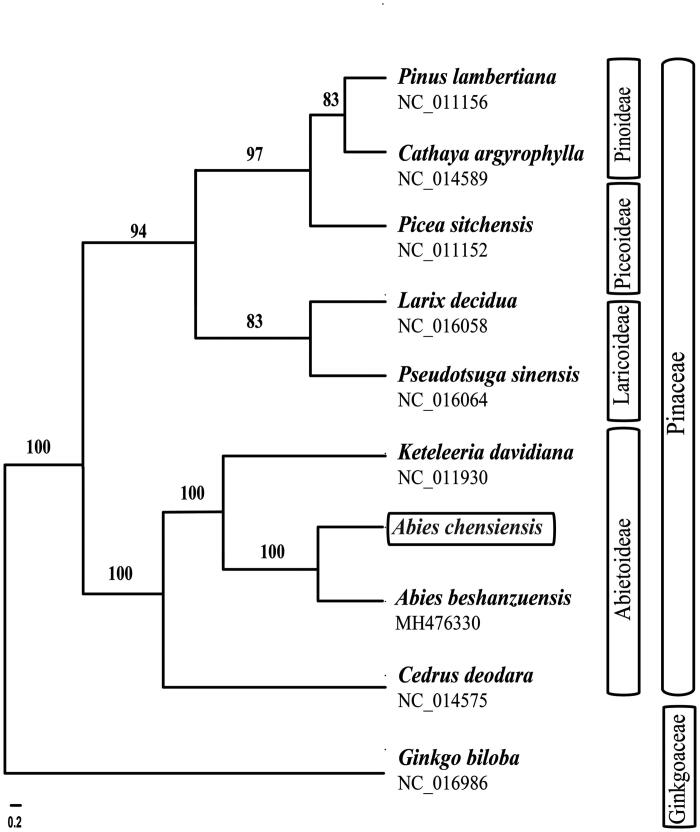
The best Maximum likelihood (ML) phylogram inferred from ten chloroplast genomes in Pinaceae and Ginkgoaceae (bootstrap value are indicated on the branches).
